# Global status of antimicrobial resistance among environmental isolates of *Vibrio cholerae* O1/O139: a systematic review and meta-analysis

**DOI:** 10.1186/s13756-022-01100-3

**Published:** 2022-04-25

**Authors:** Xin-hui Yuan, Yu-mei Li, Ali Zaman Vaziri, Vahab Hassan Kaviar, Yang Jin, Yu Jin, Abbas Maleki, Nazanin Omidi, Ebrahim Kouhsari

**Affiliations:** 1 The First Hospital of Lanzhou University, Lanzhou, 730000 China; 2 Nanjing Children’s Hospital Affiliated to Nanjing Medical University, Nanjing, 210008 China; 3 Department of Genetics, Faculty of Advanced Science and Technology, Tehran Medical Science, Islamic Azad University, Tehran, Iran; 4 Clinical Microbiology Research Center, Ilam University of Medical Sciences, Ilam, Iran; 5 Laboratory Sciences Research Center, Faculty of Paramedical Sciences, Golestan University of Medical Sciences, Gorgan, Iran; 6 Department of Laboratory Sciences, Faculty of Paramedicine, Golestan University of Medical Sciences, Gorgan, Iran

**Keywords:** Antibiotic resistance, Environmental *V. cholerae* O1/O139, Systematic review and meta-analysis

## Abstract

**Background:**

*Vibrio cholerae* O1/O139 were the predominant circulating serogroups exhibiting multi-drug resistance (MDR) during the cholera outbreak which led to cholera treatment failures.

**Objective:**

This meta-analysis aimed to evaluate the weighted pooled resistance (WPR) rates in *V. cholerae* O1/O139 isolates obtained from environmental samples.

**Methods:**

We systematically searched the articles in PubMed, Scopus, and Embase (until January 2020). Subgroup analyses were then employed by publication year, geographic areas, and the quality of studies. Statistical analyses were conducted using STATA software (ver. 14.0).

**Results:**

A total of 20 studies investigating 648 environmental *V. cholerae* O1/O139 isolates were analysed. The majority of the studies were originated from Asia (n = 9). In addition, a large number of studies (n = 15 i.e. 71.4%) included in the meta-analysis revealed the resistance to cotrimoxazole and ciprofloxacin. The WPR rates were as follows: cotrimoxazole 59%, erythromycin 28%, tetracycline 14%, doxycycline 5%, and ciprofloxacin 0%. There was increased resistance to nalidixic acid, cotrimoxazole, furazolidone, and tetracycline while a decreased resistance to amoxicillin, ciprofloxacin, erythromycin, chloramphenicol, ampicillin, streptomycin, and ceftriaxone was observed during the years 2000–2020. A significant decrease in the doxycycline and ciprofloxacin-resistance rates in *V. cholerae* O1/O139 isolates was reported over the years 2011–2020 which represents a decrease in 2001–2010 (*p* < 0.05).

**Conclusions:**

Fluoroquinolones, gentamicin, ceftriaxone, doxycycline, kanamycin, and cefotaxime showed the highest effectiveness and the lowest resistance rate. However, the main interest is the rise of antimicrobial resistance in *V. cholerae* strains especially in low-income countries or endemic areas, and therefore, continuous surveillance, careful appropriate AST, and limitation on improper antibiotic usage are crucial.

**Supplementary Information:**

The online version contains supplementary material available at 10.1186/s13756-022-01100-3.

## Introduction

Globally, WHO reported about 2.9 million new cases of cholera in 69 cholera-endemic countries and 21,000–143,000 cholera-associated deaths to occur every year worldwide [1]. Also, more than one million new cases and 5654 deaths were reported from 34 countries [2]. Two serogroups, O1 and O139 were related to the cholera outbreak and also were the predominant circulating serogroups exhibiting multi-drug resistance during the cholera outbreak [2, 3].

Over the years, some antimicrobials such as tetracyclines and fluoroquinolones have been excellently active against cholera-associated strains [4–7]. However, recently, cholera treatment failures are frequently observed with the recurrent emergence of resistant strains [4]. So, there has been a rising concern about the development of antimicrobial resistance in *V. cholerae* strains especially in low-income countries [4]. So, a comprehensive and elucidated resistance rate data is essential. Since, *V. cholerae* is a primarily/natural inhabitant of aquatic environment ecosystems worldwide, aquatic environment as the reservoir of toxigenic *V. cholerae* contribute significantly to variation and transmission in cholera epidemics [8, 9]. Nevertheless, the epidemiological impact of environmental *V. cholerae* strains is not clearly understood. To answer this vexing question, we conducted this systematic review and meta-analysis to provide extensive and elaborated data on the antimicrobial resistance patterns of environmental *V. cholerae* isolates against commonly used antimicrobials, across various regions over different periods.

## Methods

### Guidelines

This review is reported accordant with the Preferred Reporting Items for Systematic Reviews and Meta Analyses guidelines (PRISMA) [10].

### Search strategy

We systematically searched for relevant studies in PubMed, Scopus, and Embase (Until January 2020) by using the related keywords; (“*Vibrio cholerae*” OR “*V. cholerae*”) AND (“Antibiotic resistance” OR “Antimicrobial resistance”) in the Title/Abstract/Keywords fields. No limitation was used while searching databases, but the inclusion of the study in our full analysis required at least the abstract to be available in English. The search strategy was designed and conducted by study investigators. The records found through database searching were merged and the duplicates were removed using EndNote X8 (Thomson Reuters, New York, NY, USA).

### Study selection

One of the research teams (N.O.) randomly evaluated the search results and confirmed that no relevant study had been ignored. All these steps were done by the three authors (H.K, N.O., A.M.), and any disagreements about article selection were resolved through discussion, and a fourth author (E.K.) acted as arbiter. Three reviewers (H.K., N.O., A.M.) screened all titles and abstracts separately and excluded irrelevant or duplicate articles first. Three reviewers then independently evaluated the remaining articles for inclusion. Discrepancies were resolved by discussion.

### Eligibility criteria

The following items were extracted from each included study: the first author, year published, continent, country, number of environmental *V. cholerae* O1/O139 isolates, number of resistant environmental *V. cholerae* O1/O139 isolates, antibiotic susceptibility testing methods (AST; disk diffusion, agar dilution, microbroth dilution, E-test), and interpretation of resistance. The exclusion criteria were as follows: (1) studies that contained redundant data or were overlapping articles; (2) those which presented clinical or non O1/O139 *V. cholerae* isolates, animal research, reviews, meta-analysis and/or systematic review, and conference abstracts; (3) those in which resistance rates were not presented or reported; and (4) articles that included fewer than 5 V*. cholerae* O1/O139 isolates.

### Quality assessment process

The quality of the included studies was assessed by two reviewers (H.K., N.O.) separately using an adapted version of the tool proposed by the Newcastle–Ottawa assessment scale adapted for cross-sectional studies [11]. A score ranging from 0 to 7 points was attributed to each study (≥ 6 points: high quality, ≤ 5 points: low quality). A higher score indicated a higher study quality. A third reviewer (E.K) adjudicated in any cases where there was disagreement.

### Publication bias

Publication bias was analysed using Egger’s linear regression test.

### Statistical analysis

Those studies presenting raw data on antibiotic susceptibility in environmental *V. cholerae* O1/O139 isolates were included in the meta-analysis that have performed pool computing pool using a random-effects model with Stata/SE software, v.14.1 (StataCorp, College Station, TX). The inconsistency across studies was examined by the forest plot as well as the I^2^ statistic. Values of I^2^ (25%, 50%, and 75%) were interpreted as the presence of low, medium, or high heterogeneity, respectively. So, the DerSimonian and Laird random-effects models were used [11]. Subgroup analyses were then employed by publication year, geographic areas, and the quality of studies. Publication bias was assessed using Egger’s test. All statistical interpretations were reported on a 95% confidence interval (CI) basis.

### Study outcomes

The main outcome of interest was the weighted pooled resistance rate (WPR) of environmental *V. cholerae* O1/O139 to antibiotics. A subgroup analysis was performed; The subgroup analysis was then employed by (1) publication date (2001–2010, and 2011–2020), (2) continents, and (3) the quality of studies.

## Results

### Systematic literature search

**Fig. 1:**
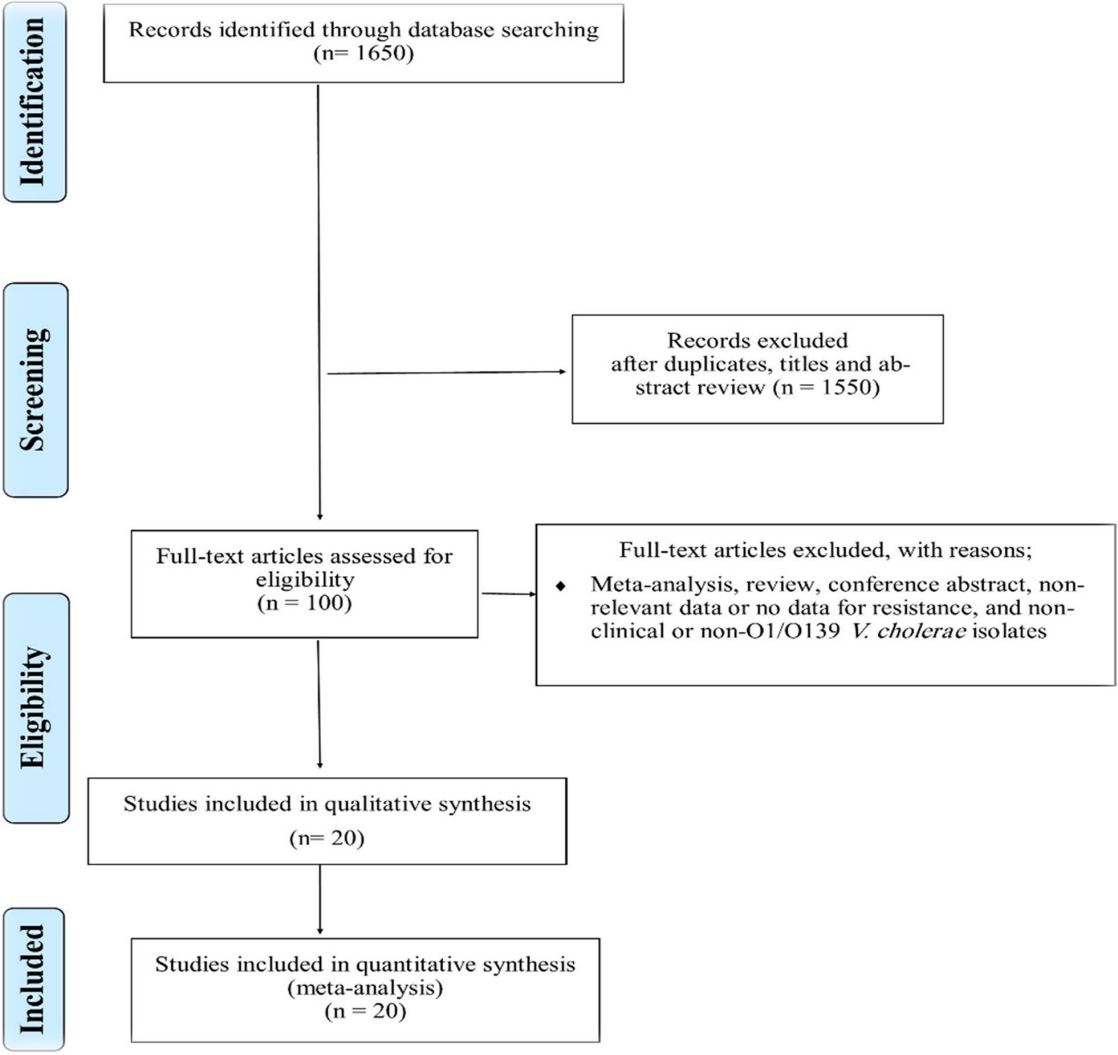
Flow chart of study selection

A total of 1650 records were identified in the initial search. From these, 1550 articles were excluded after an initial screening of the title and abstract due to their irrelevancy and redundancy. The full texts of the remaining 85 articles were reviewed (Fig. 1). Of the 85 articles, 64 were excluded for the following reasons: being meta-analysis, review, conference abstract, or not containing relevant, clinical, resistance data, or O1/O139 *V cholerae* clinical isolates. Finally, 20 cross-sectional studies [12–31] were included in this meta-analysis (Additional file 1). The studies included in this meta-analysis evaluated antibiotic resistance to ciprofloxacin, erythromycin, furazolidone, tetracycline, doxycycline, chloramphenicol, nalidixic acid, cotrimoxazole, ampicillin, streptomycin, gentamicin, ceftriaxone, and norfloxacin.

### Characteristics of included studies

**Fig. 2:**
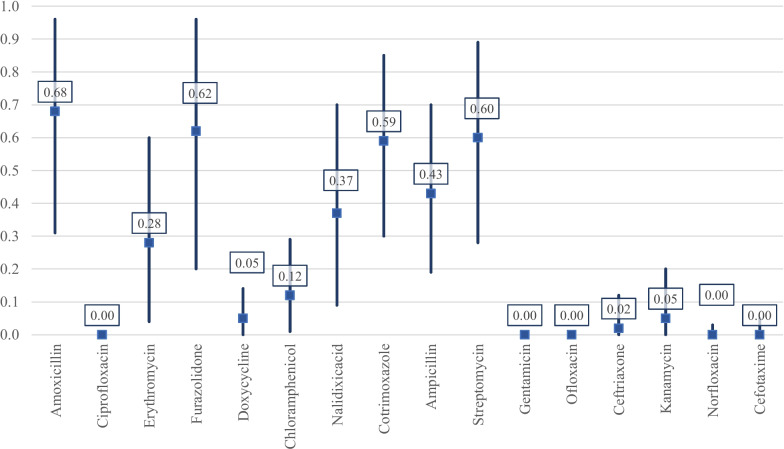
The WPRs of environmental *V. cholerae* O1/O139 isolates to antibiotics

All 21 studies included in the analysis were performed in 11 countries and had investigated 648 O1/O139 *V. cholerae* environmental isolates obtained from the drinking water, streams, storage tanks, wells, and seafoods. The majority of the studies were originated from Asia (n = 9) and Africa (n = 8), followed by America (n = 3). Disk diffusion agar test was the most common antimicrobial susceptibility testing method (n = 19), followed by a combination of two methods (agar dilution & disk diffusion agar) (n = 1). All 21 studies had a cross-sectional design. The majority of the studies (n = 15 i.e. 71.4%) included in the meta-analysis revealed the resistance to cotrimoxazole and ciprofloxacin. The WPR rates of each antibiotic and the subgroup analyses by year, continent, and the quality are shown in the Supplementary Table. The WPR rates of environmental *V. cholerae* O1/O139 isolates to antibiotics shown in Fig. 2.

### Tetracyclines-resistance

#### Tetracycline

The susceptibility to tetracycline was determined in 12 studies that included 285 environmental O1/O139 *V. cholerae* isolates; the WPR was 14% (95% CI 3–29) with substantial heterogeneity (I^2^ = 91.39%) (Table 1). We performed a subgroup analysis for two periods (2001–2010, and 2011–2020), to analyze the trends for changes in the prevalence of tetracycline resistance in more recent years (Table 1). The subgroup analysis that compared the data during 2001–2010 (WPR 13%; 95% CI 3–26) and 2011–2020 (WPR 14%; 95% CI 1–36) indicated an increase in the resistance rate. The highest resistance rate was reported in Africa, (4%, 95% CI 0–21) (Table 1).

**Table 1:** Prevalence of antibiotic-resistant in *V. cholerae* isolates from environmental samples based on years, quality, and continents

Study	Effect Size				Heterogeneity				Test of ES = 0			
	Proportion	LCI	HCI	% Weight	chi^2^	*df*	*p*	I^2^	z	*p*	Between sub-groups	Egger
Amoxicillin	0.68	0.31	0.96	100	25.55	2	0	92.17	4.65	0		0.622
2011–2020	0.58	0.45	0.71	66.45		1			11.7	0		
2001–2010	0.81	0.64	0.93	33.55		0			11.67	0		
HQ	0.58	0.45	0.71	66.45		0			11.7	0		
LQ	0.81	0.64	0.93	33.55		1			11.67	0		
Ciprofloxacin	0	0	0	100	15.29	14	0.36	8.41	0	1		0.465
2011–2020	0	0	0	65.8	2.22	8	0.97	0	0	1	0.03	
2001–2010	0.02	0	0.07	34.2	7.15	5	0.21	0.3009	1.42	0.16		
Africa	0	0	0.03	45.87	7.27	6	0.3	0.1742	0.88	0.38	0.67	
Asia	0	0	0.03	35.01	5.49	4	0.24	0.2716	0	1		
America	0	0	0.02	19.11		2		.%	0	1		
HQ	0	0	0	91.48	14.01	13	0.37	0.0722	0	0.16	0.3	
LQ	0.03	0	0.16	8.52		0		.%	1.42	1		
Erythromycin	0.28	0.04	0.6	100	24.64	4	0	83.77	2.75	0.01		0.546
2011–2020	0.2	0	0.64	76.92	20.46	3	0	0.8534	1.62	0.1	0.21	
2001–2010	0.5	0.32	0.68	23.08		0		%	7.96	0		
Africa	0.44	0.07	0.84	60.05		2		%	2.84	0	0.14	
Asia	0.12	0.01	0.28	39.95		1		%	2.6	0.01		
HQ	0.2	0	0.64	76.92	20.46	3	0	0.8534	1.62	0.10	0.21	
LQ	0.5	0.32	0.68	23.08		0		%	7.96	0		
Furazolidone	0.62	0.2	0.96	100	187.93	6	0	96.81	3.65	0		0.894
2011–2021	0.69	0.59	0.79	29.5		1		%	16.62	0	0.69	
2001–2011	0.6	0.13	0.98	70.5	83.94	4	0	0.9523	3.04	0		
Africa	0.77	0.14	1	44.17		2		%	2.88	0	0	
Asia	0.97	0.78	1	26.98		1		%	8.86	0		
America	0.16	0.07	0.27	28.85		1		%	4.71	0		
HQ	0.55	0.1	0.96	85.34	163.58	5	0	0.9694	13.75	0	0.07	
LQ	0.94	0.79	0.99	14.66		0		%	2.9	0		
Tetracycline	0.14	0.03	0.29	100	91.39	11	0.00	87.96	3.24	0.00		0.467
2011–2021	0.14	0.01	0.36	74.17	87.55	8	0.00	90.86	2.40	0.02		
2001–2011	0.13	0.03	0.26	25.83	91.39	2	0.00		3.47	0.02		
Asia	0.04	0.00	0.21	23.44		2			1.11	0.27	0.50	
Africa	0.22	0.02	0.53	51.16	79.22	5	0.00	93.69	2.49	0.01		
America	0.11	0.00	0.30	25.40		2			2.14	0.03		
HQ	0.14	0.03	0.29	100	91.39	11	0.00	87.96	3.24	0.00		
Doxycycline	0.05	0	0.14	100	22.93	6	0	73.83	1.95	0.05		0.577
2011–2021	0.03	0	0.1	72.35	8.69	4	0.07	0.5397	1.56	0.12	0.02	
2001–2011	0.17	0.07	0.3	27.65		1		%	4.43	0		
Africa	0.07	0	0.21	74.76	20.02	4	0	0.8002	2.11	0.03	0.18	
America	0	0	0.07	25.24		1		%	0	1		
HQ	0.28	0.14	0.47	15.81	8.9	5	0.11	0.4382	1.55	0.12	0	
LQ	0.02	0	0.08	84.19		0		%	5.46	0		
Chloramphenicol	0.12	0.01	0.29	100	114.33	9	0	91.25	2.63	0.01		0.401
2011–2022	0.03	0	0.11	54.31	11.52	5	0.04	0.5661	1.96	0.05	0.16	
2001–2012	0.27	0	0.71	45.69	88.58	4	0	0.9548	2.07	0.04		
Africa	0.11	0.01	0.25	64.21		0		%	2.77	0.01	0	
Asia	1	0.74	1	8.56	37.47	6	0	0.8399	9.12	0		
America	0	0	0.02	27.23		2		%	0	1		
HQ	0.09	0	0.25	90.47	88.87	9	0	0.8987	2.23	0	0	
LQ	0.47	0.29	0.65	9.53		0		.%	7.61	0.03		
Nalidixic acid	0.37	0.09	0.7	100	256.8	11	0	95.72	3.34	0		0.527
2011–2022	0.49	0.05	0.94	58.09	189.52	6	0	0.9683	2.54	0.01	0.29	
2001–2012	0.19	0.02	0.45	41.91	28.49	4	0	0.8596	2.54	0.01		
Africa	0.4	0	0.91	34.4	119.93	3	0	0.975	2.04	0.04		
Asia	0.35	0.01	0.81	40.08	37	4	0	0.8919	2.14	0.03		
America	0.37	0	1	25.52		2		%	1.24	0.21		
HQ	0.39	0.08	0.75	91.33		0		%	3.07	0	0.25	
LQ	0.19	0.07	0.36	8.67	254.79	10	0	0.9608	4.24	0		
Cotrimoxazole	0.59	0.3	0.85	100	306.32	14	0	95.43	5.29	0		0.74
2011–2023	0.66	0.3	0.95	52.97	120.59	7	0	0.942	4.55	0		
2001–2013	0.51	0.11	0.89	47.03	143.9	6	0	0.9583	3.16	0		
Africa	0.66	0.29	0.95	40.84	103.23	5	0	0.9516	4.49	0	0.84	
Asia	0.63	0.21	0.97	38.72	51.84	5	0	0.9035	3.65	0		
America	0.37	0	1	20.45		2		.%	1.24	0		
HQ	0.56	0.26	0.84	93.04	281.9	13	0	0.9539	13.14	0	0.02	
LQ	0.91	0.75	0.98	6.96		0		.%	4.8	0		
Ampicillin	0.43	0.19	0.7	100	328.8	12	0	96.05	4.62	0		0.096
2011–2023	0.39	0.06	0.77	57.08	251.54	7	0	0.9722	2.87	0	0.67	
2001–2013	0.5	0.2	0.79	42.92	52.37	5	0	0.9045	4.37	0		
Africa	0.52	0.24	0.8	35.86	42.31	4	0	0.9054	4.74	0		
Asia	0.51	0.03	0.98	42.51	220.15	5	0	0.9773	2.3	0.02		
America	0.17	0	0.58	21.63		2		%	1.62	0.11		
HQ	0.49	0.28	0.71	92.41	143.09	12	0	0.9161	6.14	0	0	
LQ	0.01	0	0.04	7.59		0		%	1.42	0.16		
Streptomycin	0.6	0.28	0.89	100	208.43	10	0	95.2	4.7	0		0.995
2011–2024	0.57	0.09	0.98	54.61	150.05	5	0	0.9667	2.81	0	0.84	
2001–2014	0.64	0.21	0.97	45.39	56.94	4	0	0.9298	3.7	0		
Africa	0.42	0	1	28.64		2		%	1.48	0.14	0.76	
Asia	0.74	0.35	1	52.68	40.84	5	0	0.8776	4.53	0		
America	0.62	0.48	0.75	18.68		1		%	11.63	0		
Gentamicin	0	0	0	100	4.57	7	0.71	0	0	1		0.645
Ofloxacin	0	0	0.01	100	0.38	3	0.95	0	0	1		
Ceftriaxone	0.02	0	0.12	100	29.21	5	0	82.88	1.15	0.25		0.489
2011–2024	0	0	0	64.13	0.99	3	0.8	0	0	1	0	
2001–2014	0.16	0.08	0.26	35.87		1		%	5.61	0		
Africa	0.04	0	0.1	36		1			2.24	0.03	0	
Asia	0	0	0	45.19		2			0	1		
America	0.14	0.05	0.29	18.8		0			4.21	0		
HQ	0.04	0	0.16	78.95	15.36	4	0	0.7395	1.48	0.14	0.03	
LQ	0	0	0.03	21.05		0		%	0	1		
Kanamycin	0.05	0	0.2	100	4.27	2	0.12	53.11	1.48	0.14		0.967
Norfloxacin	0	0	0.03	100	2.69	3	0.44	0	0	1		0.795
Cefotaxime	0	0	0.05	100	2.89	2	0.24	30.86	0.44	0.66		0.549
Amikacin	0.2	0	0.69	100	20.85	2	0	90.41	1.41	0.16		0.81

#### Doxycycline

The susceptibility to doxycycline was determined in 7 studies that included 173 O1/O139 *V. cholerae* isolates; the WPR was 5% (95% CI 0–14) with substantial heterogeneity (I^2^ = 73.83%) (Table 1). As shown in Table 1, the prevalence of doxycycline-resistance significantly decreased from 17% (95% CI 7–30) of 48 strains between 2001 and 2010 to 3% (95% CI 0–10) of 125 strains in 2011–2020, thus, the frequency of O1/O139 *V. cholerae* during the years 2011–2020 represents a > fivefold decrease over the years 2001–2010 (*p* = 0.02). The prevalence of doxycycline resistance was 7% (95% CI 0–21) among 129 isolates in Africa, and 0% (95% CI 0–7) among 44 isolates in America.

### Sulfonamides-resistance

#### Cotrimoxazole

The susceptibility to cotrimoxazole was determined in 15 studies that included 1624 O1/O139 *V. cholerae* isolates; the WPR was 59% (95% CI 30–85) with substantial heterogeneity (I^2^ = 95.43%) (Table 1). The prevalence of cotrimoxazole-resistance increased from 51% (95% CI 11–89) of 764 strains during 2001–2011 to 66% (95% CI 30–95) of 860 strains in 2012–2020. The prevalence of cotrimoxazole resistance was 66% (95% CI 29–95) among 663 isolates in Africa, 63% (95% CI 21–97) among 629 isolates in Asia, and 37% (95% CI 0–100) among 332 isolates in America.

### Fluoroquinolones

#### Ciprofloxacin

The susceptibility to ciprofloxacin was determined in 15 studies that included 376 O1/O139 *V. cholerae* isolates; the WPR was 0% (95% CI 0–0) (Table 1). The subgroup analysis that compared the data over the years 2001–2010 (WPR 2%; 95% CI 0–7) and 2011–2020 (WPR 0%; 95% CI 0–0) indicated a decrease in the resistance rate and, this difference was statistically significant (*P* = 0.03; Table 1).

#### Norfloxacin

The susceptibility to norfloxacin was determined in 4 studies that included 64 O1/O139 *V. cholerae* isolates; the WPR was 0% (95% CI 0–3) (Table 1).

#### Ofloxacin

The susceptibility to ofloxacin was determined in 4 studies that included 123 O1/O139 *V. cholerae* isolates; the WPR was 0% (95% CI 0–1) (Table 1).

### Aminoglycosides-resistance

#### Erythromycin

The susceptibility to erythromycin was determined in 5 studies that included 71 O1/O139 *V. cholerae* isolates; the WPR was 28% (95% CI 4–60) with substantial heterogeneity (I^2^ = 83.77%) (Table 1). The prevalence of erythromycin-resistance gradually increased from 50% (95% CI 32–68) of 16 strains during 2001–2010 to 20% (95% CI 0–64) of 55 strains in 2011–2020 (Table 1). Thus, the frequency of erythromycin-resistant strains during the years 2011–2020 represents a 2.5-fold decrease over the years 2001–2010. The prevalence of erythromycin resistance was 44% (95% CI 7–84) among 43 isolates in Africa, and 12% (95% CI 1–28) among 28 isolates in Asia (Table 1).

#### Streptomycin

The susceptibility to streptomycin was determined in 11 studies that included 201 O1/O139 *V. cholerae* isolates; the WPR was 60% (95% CI 28–89) with substantial heterogeneity (I^2^ = 95.2%) (Table 1). The prevalence of streptomycin-resistant strains gradually decreased from 64% (95% CI 68–92) of 91 strains during 1980–2000 to 57% (95% CI 62–88) of 110 strains in 2001–2010 (Table 1). The prevalence of streptomycin resistance was 42% (95% CI 0–100) among 58 isolates in Africa, 62% (95% CI 48–75) among 38 isolates in America, and 74% (95% CI 35–100) among 106 isolates in Asia (Table 1).

#### Gentamicin

The susceptibility to gentamicin was determined in 8 studies that included 247 V*. cholerae* O1/O139 isolates; the WPR was 0% (95% CI 0–0) (Table 1).

### Furazolidone- resistance

The susceptibility to furazolidone was determined in 7 studies that included 185 V*. cholerae* O1/O139 isolates; the WPR was 62% (95% CI 20–96) with substantial heterogeneity (I^2^ = 96.81%) (Table 1). The subgroup analysis that compared the data during 2001–2010 (WPR 6%; 95% CI 13–98) and 2011–2020 (WPR 69%; 95% CI 59–79) indicated an increase in the resistance rate (Table 1). The highest prevalence of furazolidone resistance was 97% (95% CI 78–100) among 50 isolates in Asia (Table 1).

### Chloramphenicol-resistance

The susceptibility to chloramphenicol was determined in 10 studies that included 244 O1/O139 *V. cholerae* isolates; the WPR was 12% (95% CI 1–29) with substantial heterogeneity (I^2^ = 91.25%) (Table 1). The subgroup analysis compared the data during 2001–2010 (WPR 27%; 95% CI 0–71) and 2011–2020 (WPR 3%; 95% CI 0–11) (Table 1). Thus, the frequency of chloramphenicol-resistance during the years 2011–2020 represents a ninefold decrease over the years 2001–2010. The highest resistance rate was reported in Asia, followed by Africa (100%, 95% CI 74–100; 11%, 95% CI 1–25) (Table 1).

### Nalidixic acid-resistance

The susceptibility to nalidixic acid was determined in 12 studies that included 249 O1/O139 *V. cholerae* isolates; the WPR was 37% (95% CI 9–70) with substantial heterogeneity (I^2^ = 95.72%) (Table 1). The subgroup analysis compared the data during 2001–2010 (WPR 19%; 95% CI 2–45) and 2011–2020 (WPR 49%; 95% CI 5–94) (Table 1). Thus, the frequency of nalidixic acid- resistance during the years 2011–2020 represents a ~ 2.5-fold increase over the years 2001–2010. The highest resistance rate was reported in America (37%, 95% CI 0–100).

### β-Lactams-resistance

#### Ampicillin

The susceptibility to ampicillin was determined in 13 studies that included 379 O1/O139 *V. cholerae* isolates; the WPR was 43% (95% CI 19–70) with substantial heterogeneity (I^2^ = 96.05%) (Table 1). The subgroup analysis compared the data during 2001–2010 (WPR 50%; 95% CI 20–79) and 2011–2020 (WPR 39%; 95% CI 6–77) indicated a decrease in the resistance rate. The highest resistance rate was reported in Europe followed by Asia, Africa, and America (51%, 95% CI 3–98; 52%, 95% CI 24–80; 17%, 95% CI 0–58).

#### Ceftriaxone

The susceptibility to ceftriaxone was determined in 15 studies that included 1851 O1/O139 *V. cholerae* isolates; the WPR was 12% (95% CI 2%-27%) with substantial heterogeneity (I^2^ = 97.99%) (Table 1). The subgroup analysis compared the data during 2001–2010 (WPR 24%; 95% CI 1–60), and 2011–2020 (WPR 5%; 95% CI 0–18) (Table 1). Thus, the frequency of ceftriaxone-resistance during the years 2011–2020 represents a 4.8-fold decrease over the years 2001–2010. The highest resistance rate was reported in Africa, followed by Europe (43%, 95% CI 0–100; 8%, 95% CI 2–20). A significant difference was found in the methods used for AST (*p* = 0.02).

#### Publication bias

Begg’s and Egger’s regression tests were performed to assess publication bias. The shapes of the funnel plots do not show obvious evidence of asymmetry. However, the *p*. value of Egger’s test confirmed the existence of publication bias for all the WPRs evaluated [(A) ciprofloxacin, *p* = 0.465; (B) erythromycin, *p* = 0.546; (C) furazolidone, *p* = 0.894; (D) tetracycline, *p* = 0.467; (E) doxycycline, *p* = 0.577; (F) chloramphenicol, *p* = 0.401; (G) nalidixic acid, *p* = 0.527; (H) cotrimoxazole, *p* = 0.74; (I) amoxicillin, *p* = 0.622; (J) streptomycin, *p* = 0.995; (K) gentamicin, *p* = 0.645; (L) ceftriaxone, *p* = 0.489; (M) ampicillin, *p* = 0.096; (N) norfloxacin, *p* = 0.795; (O) kanamycin, *p* = 0.967; (P) cefotaxime, *p* = 0.549; and (Q) amikacin, *p* = 0.81] (Table 1).

## Discussion

This systematic review and meta-analysis was conducted to consider the global prevalence of antibiotic resistance in environmental *V. cholerae* O1/O139 isolates. It is significant to obtain more data about the resistance profiles of circulating environmental *V. cholerae* O1/O139 strains. Cholera as an ancient and acute infectious disease is considered a major public health issue primarily in developing countries [32]. The antimicrobial resistance is increasing in *V. cholerae* isolates over recent years and has become a major risk to cholera treatment strategies [4]. Thus, a comprehensive and potent policy is required to control and treat cholera.

Tetracyclines has long been the most effective antibiotic class for cholera treatment. Nevertheless, previously published studies reported an increase in tetracycline-resistant strains of *V. cholerae* worldwide [33, 34]. This meta-analysis revealed that resistance status to tetracycline and doxycycline in environmental *V. cholerae* O1/O139 isolates were 14% and 5%, respectively. We found that the tetracycline-resistance rate of *V. cholerae* isolates was considerably varying in different geographical areas. The regional divergences in tetracycline-resistance rate of *V. cholerae* isolates may result from infection control policies, antibiotic stewardship, the lack of a uniform consumption pattern, exposure to the same antibiotics in different regions, and various AST methods. Our meta-analysis presented that trend of tetracycline-resistance had a minor increase from 2001–2010 to 2011–2020 (13% and 14%). Also, the frequency of doxycycline resistant O1/O139 *V. cholerae* isolates shows, fivefold decrease over the years 2001–2010. Additionally, the frequency of tetracycline and doxycycline-resistant isolates in Africa is more to compare with Asia and America. The tetracycline-resistance determinants may be developed, transferred and exchanged between environmental and clinical *V. cholera* isolates through the horizontal gene transfer mechanisms, the active efflux of antibiotics from the bacterial cell, the production of ribosomal protection proteins (encoded by *tet* genes), target site mutation, decreased drug permeability, and enzymatic degradation of the antibiotics [35, 36]. Recently, a systematic review and meta-analysis [37] of tetracyclines resistance in clinical *V. cholera* O1 isolates showed higher rates than observed in our study; 20% (95% CI, 10–30) for tetracycline and 7% (95% CI, 3–10) for doxycycline. The analyses differed in the origin of the isolates since, in our analyses, the data on *V. cholera* isolates of clinical origin were not included. Three representatives of fluoroquinolones were analysed in our study; ciprofloxacin, norfloxacin, and ofloxacin. In our study, the resistance rate of environmental O1/O139 *V. cholerae* isolates against fluoroquinolones was0%. The prevalence of ciprofloxacin-resistant O1/O139 *V. cholerae* isolates shows, 2% decrement over the years 2001–2010. Our data in line with some studies [38, 39] showed that fluoroquinolones have excellent activity against *V. cholerae* species. However, fluoroquinolones-resistance in *V. cholerae* strains started rising from July 1996 [40]. Mohammed and colleagues [41] conducted a systematic review and meta-analysis to review prior data on antimicrobial resistance of *V. cholerae* from sub-Saharan Africa. They reported a huge high resistance rate (44.0%) to fluoroquinolones. However, no resistance to fluoroquinolones was reported in Miwanda study [42]. The variable levels of resistance to fluoroquinolones may have been resulted from surveillance programs, widespread consumption of these antibiotics, and different AST methods.

Although previously published study proposed erythromycin, a macrolide as a substitute to tetracyclines in young children and pregnant women [43, 44]. Our findings showed that the highest resistance rate was towards erythromycin (28%; 4%-60%). Previous studies conducted in Asia and Africa reported high rates of erythromycin-resistance in *V. cholerae* [34, 41, 43].

The use of chloramphenicol has been restricted in some areas such as India in the past due to the availability of more effective antibiotics with fewer side effects [45]. Our study revealed an absolute chloramphenicol-resistance rate (100%) in Asia. The prevalence of chloramphenicol-resistance in *V. cholerae* isolates shows, sevenfold decrease over the years 2001–2010.

Hitherto furazolidone and nalidixic acid have been commonly used for cholera therapy. But currently, because of the high resistance rate in *V. cholera*e isolates these antibiotics were less effective [46]. In the present study, a high resistance rate of *V. cholera* against furazolidone (88%) and nalidixic acid (37%) were found. Yousefi et al., in another meta-analysis on Iranian isolates, claimed similar findings [33]. A significant increase in furazolidone-resistance might be related to the increased consumption of this antibiotic in low-income countries [46].

Trimethoprim/Sulfamethoxazole (cotrimoxazole) was frequently referred as antibiotic for gastroenteritis therapy. Nevertheless, in our study, a high resistance rate against trimethoprim/sulfamethoxazole was found (59%) among environmental *V. cholerae* strains, and this result is in line with the other studies [47–49]. Therefore, the rapid and reliable diagnosis between *V. cholerae* and other causative agents of gastroenteritis helps us to apply appropriate options in cholera therapy [49].

The main cause of shedding *V. cholera* to environments is the consumption of wastewater and human excreta for farming or in the aquaculture systems [50, 51]. While, antibiotic excretion from urine and faeces of humans or farm animals, and/or disposal of antibiotics may lead to the development of resistant strains or treatment failures [46, 50, 51].

## Conclusions

This meta-analysis has provided the major insights into the epidemiologically antibiotic resistance pattern of environmental *V. cholera* in three periods (1980–2000, 2001–2010 and 2011–2020) and has emphasized the distribution of antibiotic-resistant strains in continents. Our meta-analysis showed a low resistance rate against some antibiotics, including fluoroquinolones, gentamicin, ceftriaxone, doxycycline, kanamycin, and cefotaxime. However, antimicrobial resistance is on the increasing slope, and the main interest is the rise of antimicrobial resistance in *V. cholerae* strains especially in low-income countries or endemic areas. Finally, to control the development and the increase of resistant strains, continuous surveillance, careful and appropriate AST and limitation on improper antibiotic usage are crucial.

## Supplementary Information

**Additional file 1** The characteristics of cross-sectional studies included in this meta-analysis.

## Data Availability

All the data in this review are included in the manuscript.

## Abbreviations

MDR: Multidrug-resistant
WPR: Weighted pooled resistance
AST: Antibiotic susceptibility testing
PRISMA: Preferred reporting items for systematic reviews and meta analyses guidelines
CI: Confidence interval
